# It is time to progress beyond the System 1 versus System 2 dichotomy

**DOI:** 10.1007/s40037-015-0202-z

**Published:** 2015-07-17

**Authors:** Jerome I. Rotgans

**Affiliations:** National Institute of Education, Nanyang Technological University, 1 Nanyang Walk, 637616 Singapore, Singapore

There is no doubt that diagnostic reasoning is at the heart of the medical profession. Being able to generate a correct diagnosis is, however, not always easy and mistakes are common. So common that according to some sources 70 % of malpractice claims can be ascribed to physician negligence and diagnostic error [1]. As a consequence, medical education has focused on the assessment of diagnostic reasoning in an attempt to detect flaws in reasoning and correct them. Over the years a variety of elaborate assessment formats were introduced, such as key feature problems, script concordance tests, mini clinical evaluation exercises, and the standardized direct observation tool, to name a few. The purpose of these assessments is to gain more insights into the process of diagnostic reasoning itself rather than simply looking at the ‘end product’ as is the case when using more conventional assessment formats such as multiple-choice question (MCQ) tests [2]. Despite the fact that MCQ tests are routinely used for high-stake medical examinations, a common concern of using them is that only the end product is measured (i.e., the right answer is selected) without the teacher being able to gain detailed insights into the reasoning process that led to that answer. This concern, as Durning et al., demonstrate in their paper on ‘Dual processing theory and experts’ reasoning: exploring thinking on national multiple-choice questions’, is not necessarily warranted [3].

In their study Durning et al. make use of MCQs to elicit a diagnosis and then apply an innovative retrospective analysis of the diagnostic thinking processes by analysing think-aloud protocols of the participants in which they elaborate how they reached their diagnosis. The protocols were coded according to dual-process theory in which the distinction is made between the non-analytical System 1 thinking and the analytical System 2 thinking [4]. System 1 is considered a heuristic response of the mind, which is fast, relying on automatic activation of illness scripts stored in memory based on limited data from the patient (also called pattern recognition), whereas System 2 is considered slow, deliberate, and systematic. By quantifying the number of words and concepts occurring in the transcripts of the think-aloud protocols, Durning et al. were able to examine whether the diagnosis for each item was reached using System 1, System 2 or a combination of both. One major finding of the study is that neither System 1 nor System 2 alone is responsible for arriving at a correct diagnosis, but a combination of both.

This finding is important because it reminds us that more research is needed to further investigate the interaction between the two systems. Currently it appears that a polarization has taken place in the diagnostic reasoning literature in which one faction of researchers presents System 1 thinking as the hallmark of medical expertise as it is fast, intuitive, and dependent on the experience and knowledge of the physician [5], whereas the other camp defends System 2 thinking as the most appropriate diagnostic approach, as it is systematic, objective, and supposed to correct errors typically associated with System 1 thinking (e.g., premature closure and cognitive biases)[6]. Reconciliation between both camps is unlikely to happen anytime soon, despite the fact that researchers from both sides acknowledge (almost like a disclaimer typically at the beginning of introductions to their papers) that both Systems interact. However, concrete research that has demonstrated how they interact is scarce and necessary to advance the field.

What follows are three suggestions that may be worth considering when exploring this issue further. First, we need to take a closer look at how both Systems interact; it is clear that they interact, but how they do this is not well understood. For instance, Schmidt and Marmede observed in their work on deliberate reflection that the initial generation of a diagnostic hypothesis is most likely generated intuitively and automatically. Depending on the situation and level of expertise of the physician, he or she may feel the need to consider (one or) more alternative hypotheses [7]. To compare and contrast the main hypothesis with these alternative hypotheses, a more deliberate analysis is required [8]. In short, it appears that System 1 is always activated to form an initial hypothesis and System 2 to evaluate alternative options [4]. If the physician has much expertise (e.g., has seen signs and symptoms belonging to a particular diagnosis frequently before), System 2 may be less likely to be activated because there is no doubt about the diagnosis. If the physician has less expertise regarding the case, it is likely that a more deliberate and analytical evaluation of possible alternative diagnoses is required.

Second, a clarification is warranted when it comes to the issue of case difficulty vs. expertise. Frequently, case difficulty (or in the current paper MCQ item difficulty) is considered a significant factor that influences diagnostic accuracy. The reasoning is straightforward; if a case is relatively more difficult it is more likely that errors occur. Other studies stress the level of expertise of the physician in diagnostic reasoning; more experienced physicians typically make less mistakes. In some studies both are even factored in (i.e., high vs. low levels of expertise and high vs. low case complexity) [9]. This distinction appears oxymoronic because case difficulty is always a function of expertise; an experienced physician would find a particular case easy to diagnose, whereas a first-year resident may have difficulties with the same case in question. The case is the same, but the level of expertise varies. Following this logic, it appears appropriate to only consider the level of expertise of the physician, rather than case difficulty in future studies.

Third and perhaps most important, we need to realize that System 1 as well as System 2 are dependent on knowledge. Both the activation of one or more initial hypotheses and the analytical assessment of their truth value are based on knowledge retrieved from memory. The difficulty with memory is that it is prone to error and distortions [10, 11]. Such errors and distortions include imagination inflation, gist-based and associative-memory errors, post-event misinformation effects, and recency effects. It would advance the field if research into clinical reasoning were less obsessed with distinctions such as those between System 1 and 2, and would focus more on how understanding memory can inform diagnostic expertise.
